# Non-genetic red blood cell variability can modulate hemoglobin consumption by intracellular *Plasmodium falciparum*

**DOI:** 10.1128/msystems.00554-26

**Published:** 2026-09-04

**Authors:** Joseph R. Perko, Abhyudai Singh, Secilia I. Lopez, Armando Estrada, Shweta Singh, Janice M. Moser, Kelly Haupfear, George J. Klarmann, Sabrina J. Tuson, Vincent B. Ho, Shirley Luckhart, Andreas E. Vasdekis

**Affiliations:** 1 Department of Physics, University of Idaho 5640, Moscow, Idaho, USA; 2 Electrical and Computer Engineering, University of Delaware 736760, Newark, Delaware, USA; 3 Department of Biological Sciences, University of Idaho 123394, Moscow, Idaho, USA; 4 4D Bio³ Center for Biotechnology, Department of Radiology and Bioengineering, Uniformed Services University of the Health Sciences 1685, Bethesda, Maryland, USA; 5 The Geneva Foundation 104849, Tacoma, Washington, USA; 6 Sciperio, Inc., Orlando, Florida, USA; 7 Department of Entomology, Plant Pathology and Nematology, University of Idaho 5640, Moscow, Idaho, USA; Yale University, New Haven, Connecticut, USA; U.S. Department of Health and Human Services, Washington, DC, USA

**Keywords:** phenotypic variability, *Plasmodium falciparum*, red blood cells, non-genetic variation, cellular noise

## Abstract

Natural, non-genetic variation among host cells can shape infection outcomes, yet its role in intracellular parasite dynamics remains poorly understood. Here, we show that variability in hemoglobin (Hb) content among individual red blood cells (RBCs) modulates nutrient consumption by *Plasmodium falciparum*, the most lethal malaria parasite. Using label-free quantitative-phase imaging (QPI), we measured cytosolic Hb in thousands of uninfected and infected RBCs across the 48-h asexual cycle. Hb content varied widely among uninfected cells, and after invasion, infected cells mirrored and amplified this distribution: variability increased from 17% in uninfected RBCs to >100% in schizonts. A minimal predator-prey model accurately reproduced the observed spread in parasite Hb consumption, showing that host variability alone can generate much of the diversity in parasite feeding rates traditionally attributed to parasite factors. These findings provide the first evidence that host phenotypic noise drives variation in Hb consumption among genetically identical parasites. Incorporating host-to-host variability into within-host models could improve predictions of parasite growth, refine the activation kinetics of Hb-dependent antimalarials, and reveal analogous principles in other intracellular infections where host variability and host-parasite crosstalk can shape pathogen outcomes. This systems-level perspective emphasizes the need to account for host heterogeneity in both experimental design and therapeutic strategies.

**IMPORTANCE:**

Cell-to-cell phenotypic variability is a fundamental feature of living systems, yet its impact on host-pathogen interactions has been largely overlooked. Here, we show that natural, non-genetic variation in red blood cell hemoglobin strongly shapes how *Plasmodium falciparum*—the most lethal malaria parasite—consumes Hb inside host cells. Using quantitative imaging and a mathematical model, we demonstrate that host variability alone can explain much of the observed differences in parasite feeding rates. This perspective reframes parasite Hb consumption as an emergent property of host-parasite interactions. More broadly, it highlights how natural variability among host cells can influence the course of intracellular infections, with potential implications for predicting pathogen growth and optimizing drug treatment strategies.

## INTRODUCTION

Non-genetic cell-to-cell phenotypic differences arise from intrinsic noise (e.g., stochastic fluctuations in gene product levels) and extrinsic factors (e.g., cell age or variation in the extracellular environment) (1–8). Such variability can strongly influence cell-to-cell variability in gene expression, stress responses, cell cycle duration, and metabolic fluxes (8–14). Because many infections start from one or just a handful of cells, even small host- or pathogen-level fluctuations early in infection can disproportionately shape disease trajectories (15). In microbiology, most attention has focused on pathogen variability, revealing bet-hedging, antibiotic persistence, and tolerance strategies (16–20). Far less is known about how natural host variability shapes intracellular infection.

In addition to shaping pathogen phenotypes, non-genetic cell-to-cell differences can affect the phenotype of the host cells that pathogens invade. For example, non-genetic variability in host receptor density, metabolic reserves, and intracellular pH can alter the probability of attachment, entry, and growth (21–26). Despite this potential, and to the best of our knowledge, the role of non-genetic host variability in infection remains considerably less understood. Narrowing this knowledge gap could advance our understanding of host-pathogen interactions and improve therapeutic strategies. Here, we focus on erythrocytic, asexual-stage *Plasmodium falciparum* and explore whether non-genetic cell-to-cell variation in hemoglobin (Hb) concentration of host red-blood-cells (RBCs) affects parasite Hb consumption.

Malaria is one of three leading global infectious diseases, along with HIV/AIDS and tuberculosis, predominantly affecting impoverished populations in sub-Saharan Africa and, to a lesser degree, in Southeast Asia (27). In these regions, infection with *P. falciparum* results in the greatest number of human deaths due to malaria (27). The *P. falciparum* life cycle begins with transmission through the bite of an infected *Anopheles* spp. mosquito, introducing the parasite into the bloodstream and initiating a complex developmental process within the host (28). Following mosquito transmission, *P. falciparum* parasites enter the liver, undergo asexual reproduction, and then emerge to invade RBCs. Within RBCs, *P. falciparum* undertakes an asexual cycle of ~48 h, during which it digests up to 80% of host cell Hb to obtain amino acids and maintain osmotic balance (29). Hb degradation also liberates free heme that chemically activates artemisinin (ART), the current frontline therapy for falciparum malaria (30–33). Thus, host-to-host variability in Hb availability has direct consequences not only for parasite physiology but also for the efficacy of antimalarial treatment.

Here, we investigated whether host cell Hb heterogeneity translates into measurable differences in parasite nutrient consumption. Using single-cell, quantitative-phase imaging (QPI), we detected significant cell-to-cell differences in the Hb concentration among uninfected RBCs. We then quantified the Hb concentration at defined post-invasion timepoints, revealing considerable variability among infected RBCs. A minimal predator-prey model showed that this pattern can be explained by the Hb variability of host RBCs. This systems-level approach revealed that natural, non-genetic variation in host RBC physiology can modulate *P. falciparum* development. While previous studies established that population-level differences, such as donor lot, storage conditions, or cell age, influence infection (34, 35), our work demonstrates that cell-to-cell phenotypic noise within a single host is sufficient to drive heterogeneity in Hb consumption. This highlights the importance of incorporating single-cell host heterogeneity into predictive models of infection and treatment.

## RESULTS

### Multimodal single-cell imaging

#### Hb quantification

**Fig 1:**
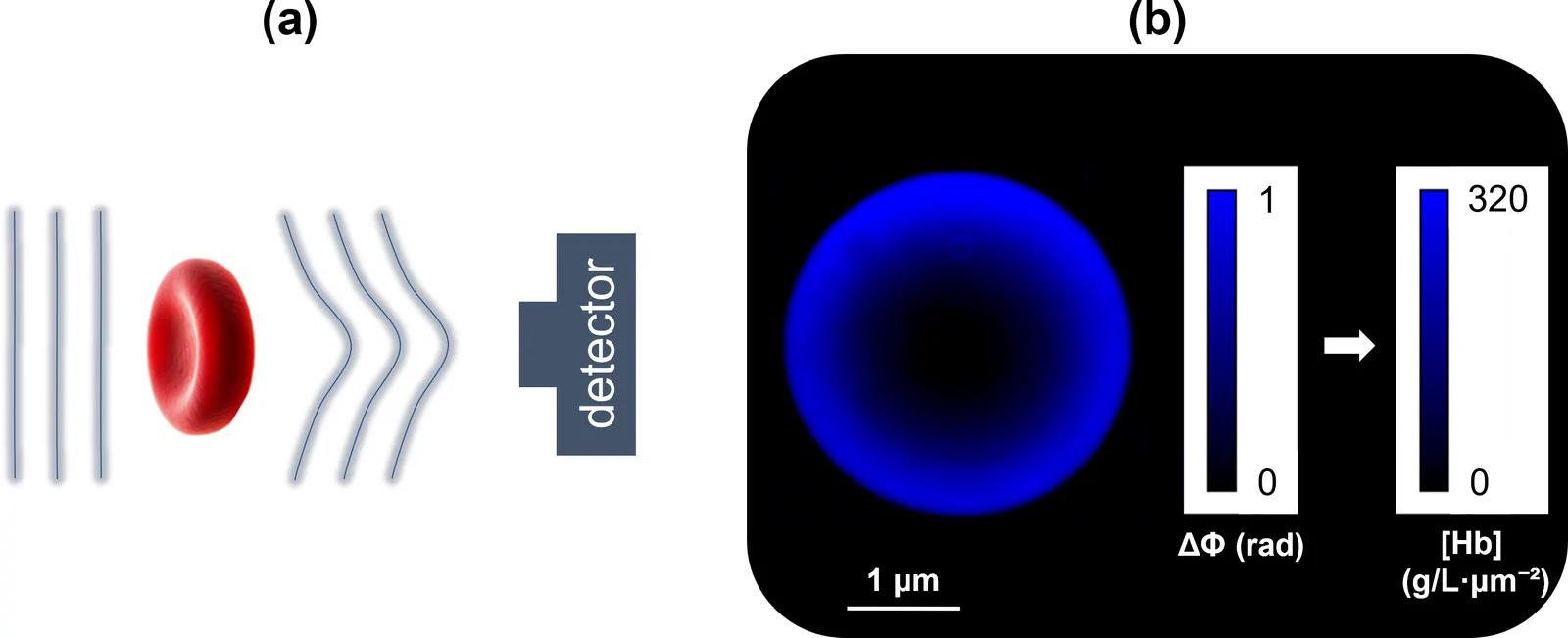
Quantitative phase imaging converts optical phase shifts into local hemoglobin (Hb) concentration in a single red blood cell. (**a**) A collimated plane wave traverses an erythrocyte, acquiring a spatially varying phase delay that encodes the cell’s internal refractive-index distribution; the distorted wavefront is captured by a camera detector in the QPI system. (**b**) A representative QPI map of a healthy RBC; scale bars on the left denote the measured wavefront distortion (ΔΦ, left) and its conversion to local Hb concentration (in g/L, right); scale bar, 1 µm.

Label-free QPI is a well-established technique for quantifying Hb concentration in uninfected and falciparum-infected RBCs (36–39). QPI records the optical-path-length shift introduced by the specimen at each pixel (Fig. 1a); in RBCs, this shift correlates with the cytosolic refractive index and therefore the protein (predominantly Hb) concentration (40). Unlike recently published, staining-based, protocols, QPI is completely label-free, thus, preserving parasite physiology (41, 42). We specifically implemented the spatial light interferometric microscopy (SLIM) variant of QPI (Materials and Methods) for its enhanced sensitivity and wide-field acquisition with improved throughput rates (43). For uninfected RBCs, we measured the average phase shift of the cytosol (ΔΦ_ave_), while for infected RBCs, we excluded parasitized regions of the cell. After this masking step, the host cell phase estimate at all stages was computed from an average of more than 4,000 pixels per cell. We quantified the Hb concentration ΔΦ_ave_ using this expression (37) (Fig. 1b):


(1) $$\left[Hb\right]=\frac{\lambda }{2\cdot \pi \cdot \alpha }\cdot \Delta \Phi_{ave}$$

equation 1 gives the areal dry-mass density of Hb (g/L·μm^−2^), referred to here as “Hb concentration,” with λ = 0.55 µm (illumination wavelength) and α = 0.2 mL/g (Hb refractive increment) (44). Through the quantification of Hb concentration and independent measurements of cell size (area), we were also able to quantify the corpuscular Hb per RBC (RBC_mass_) using the following expression (40):


(2) $$RBC_{mass}=\Delta \Phi_{ave}\cdot \frac{area_{RBC}\cdot \lambda }{2\cdot \pi \cdot \alpha }$$

#### Stage classification

To classify the life cycle stage of each parasite, we complemented the SLIM-QPI quantification of Hb with propidium-iodide fluorescence (FL) of the same infected RBCs. In this way, each cell used for stage classification had a corresponding paired QPI image and PI epifluorescence image. The QPI images provided the label-free morphological/transmitted-phase information used for staging; no separate DIC or conventional brightfield images were acquired. FL imaging enabled visualization of the parasite nucleus and determination of the parasite life cycle stage. Unlike recent QPI developments (45), the SLIM module incurs polarization-dependent optical losses (43). To minimize these losses, we collected PI epifluorescence images on an independent optical path equipped with a sensitive sCMOS sensor (Materials and Methods). To carry out stage classification, we first cropped the acquired QPI-FL image pairs to 400 × 400-pixel areas to center the infected cell and minimize background. Two trained annotators (JRP and SIL) independently classified parasites into ring (R), late ring or early trophozoite (LR-ET), trophozoite (T), and schizont (Sch) with 98% inter-rater agreement. The stage criteria were (46) as follows:

- R, ring structure featuring a thin annular cytoplasm with a single condensed nucleus (~0–14 h after invasion [47]);
- LR/ET, ring structure exhibiting a thicker ring perimeter and the beginning of cytoplasmic expansion (~14–26 h [47]);
- T, one or two enlarged nuclei, with host RBCs retaining their original biconcave shape (~26–38 h [47]);
- Sch, more than three nuclei within an RBC, but with clear, nascent merozoites evident (~38–48 h [47]).

**Fig 2:**
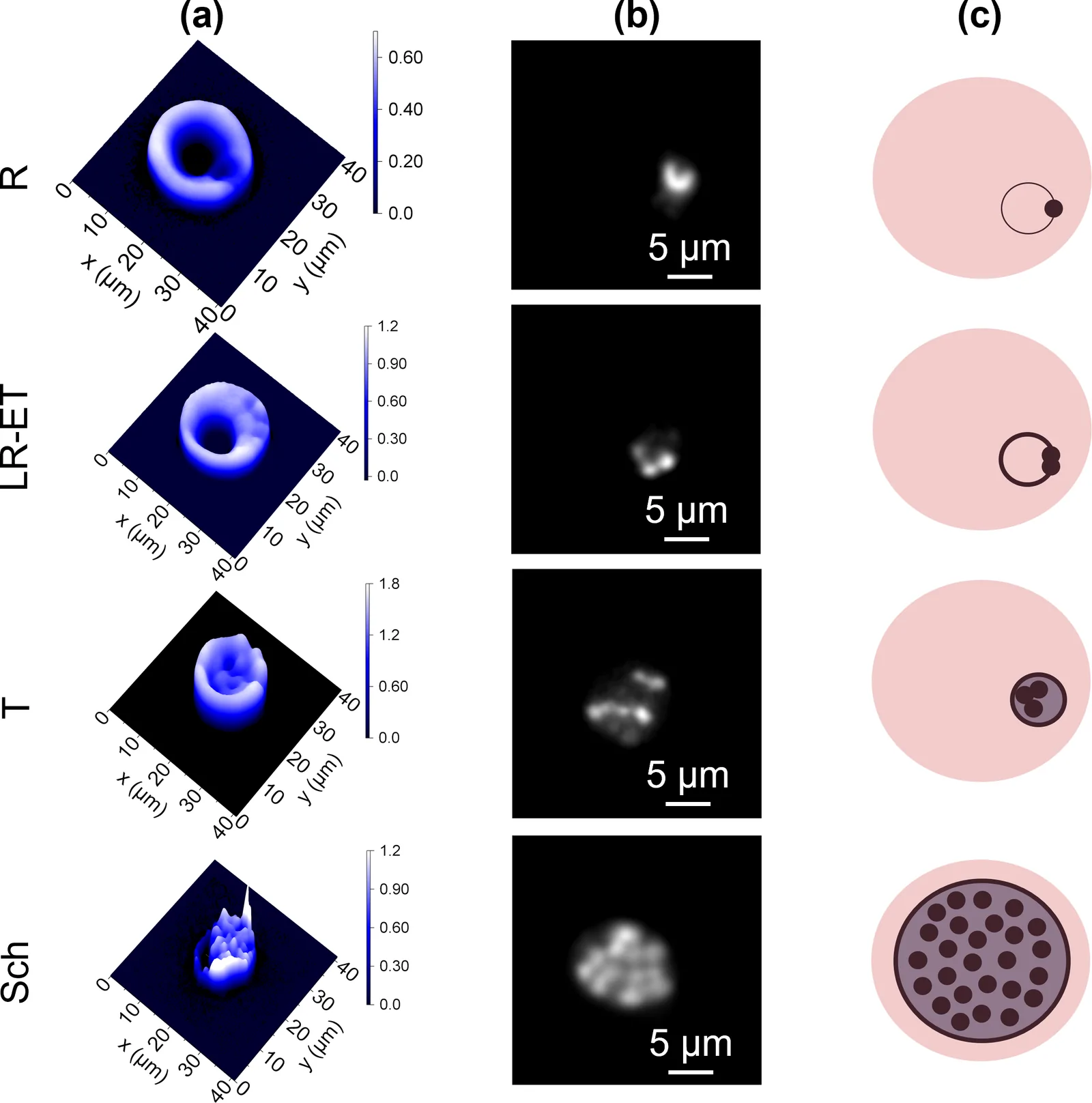
Paired quantitative-phase and PI fluorescence views of *Plasmodium falciparum*-infected RBCs over the intra-erythrocytic cycle. (**a**) QPI and (**b**) PI fluorescence maps acquired from the same infected RBCs at different life cycle stages (R, rings; LR-ET, late rings-early trophozoites; T, trophozoites; Sch, schizonts); scale bars in panel **a** denote the wavefront distortion (ΔΦ) in radians. (**c**) Schematic illustrations of idealized morphological references for each stage.

Figure 2 displays representative paired QPI and PI fluorescence images of the same infected RBCs at different life cycle stages from our data set, illustrating the progressive increase in parasite biomass and the PI-positive nuclear signal used for stage classification. Importantly, the cultures were not synchronized and were imaged randomly as they occurred naturally in continuous *in vitro* growth. Thus, each sampled stage reflects a cross-sectional ensemble of parasites rather than a synchronized cohort followed over time. In total, we classified 1,131 RBCs, including uninfected cells (*n*_RBC_ = 385) and infected ones with parasites at different life cycle stages (*n*_R_ = 70, n_LR/ET_ = 275, *n*_T_ = 307, n_Sch_ = 94). As further detailed in Materials and Methods, this distribution was imbalanced, with relatively fewer rings and schizonts. To account for unequal sample sizes, we used nonparametric bootstrapping (10,000 resamples per stage) to estimate 95% confidence intervals for both stage means and coefficients of variation, as further detailed in Materials and Methods.

### Host cell variability revealed by single-cell imaging

**Fig 3:**
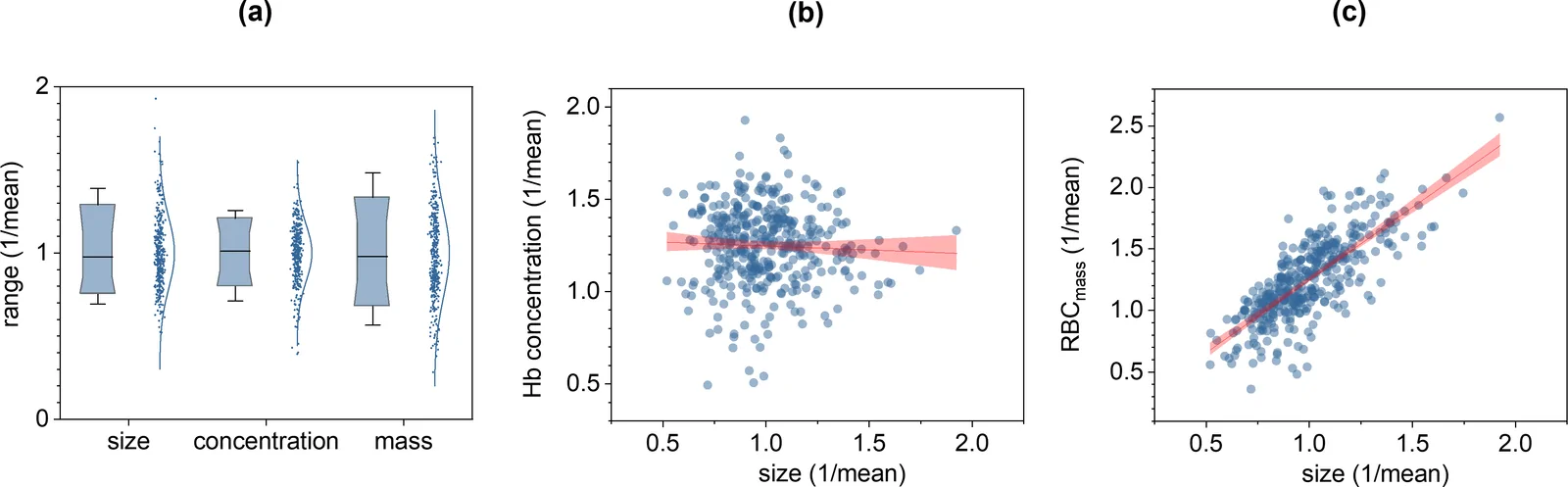
Single-cell variability in RBC size and Hb content. (**a**) RBC cell-to-cell differences in size, Hb concentration, and corpuscular Hb mass (all normalized over the mean); box charts represent 10%, 25%, 70%, and 90% ranges, while whiskers represent the 5%–95% range; dots represent the experimental data and solid lines the corresponding parametric log-normal fits to the experimental distributions. (**b**) The Hb concentration of uninfected RBCs normalized to the population mean as a function of the RBC cell size (also normalized over the mean); red line represents the linear regression analysis and its 95% confidence intervals (red shaded area); the slope was determined by bootstrapping (10,000 samples; Materials and Methods) and was found to be −0.0341 with [−0.103, 0.038] 95% confidence intervals. (**c**) Normalized cell mass versus cell size for the same RBCs as in panel **a**; a near-unity slope of 0.947 and [0.872, 1.023] 95% confidence intervals reveal a near-proportional relationship.

Using QPI, we first profiled uninfected RBCs (Materials and Methods). This data revealed a substantial cell-to-cell non-genetic differences in cell size (approximated through the projected cell area), Hb concentration, and corpuscular Hb mass of individual RBC cells (Fig. 3a; Fig. S1). We quantified these non-genetic cell-to-cell differences using the coefficient of variation (CV), which we found to be 21.5% in cell size, 17.1% in Hb concentration, and 26.4% in corpuscular Hb mass. Similar dispersion levels have been reported elsewhere in single-cell RBC studies (38), and are consistent with the “optimal-radius” distribution that is believed to maximize oxygen advection in the micro-circulation (48). Concurrently, comparable levels of non-genetic variability in corpuscular Hb mass have been reported to persist across different blood storage conditions, suggesting that this variation arises from a more intricate mechanism, as further elaborated in Discussion (38).

Trait-to-trait correlations at the single-cell level clarified the structure of this heterogeneity. Specifically, we observed no correlation between RBC size and Hb concentration (Pearson *r* = –0.04, *P =* 0.4, and slope = −0.0341 with [−0.103, 0.038] 95% confidence intervals; Fig. 3b). The lack of correlation suggests that erythrocyte size and cytosolic Hb packing are regulated largely independently during erythropoiesis. By contrast, cell size and Hb mass showed a strong positive correlation (39) (Pearson *r* = 0.77, *P <* 0.0001, and slope = 0.95 with [0.87, 1.02] confidence intervals; Fig. 3b), indicating that larger RBCs contain proportionally more total Hb mass while maintaining similar Hb concentration. This pattern is consistent with approximately conserved intracellular Hb packing across RBCs of different size (48). This quantitatively heterogeneous host landscape set the stage for examining how *P. falciparum* responds to host cell-related variation, as detailed in the following sections.

### Non-genetic differences in Hb consumption

**Fig 4:**
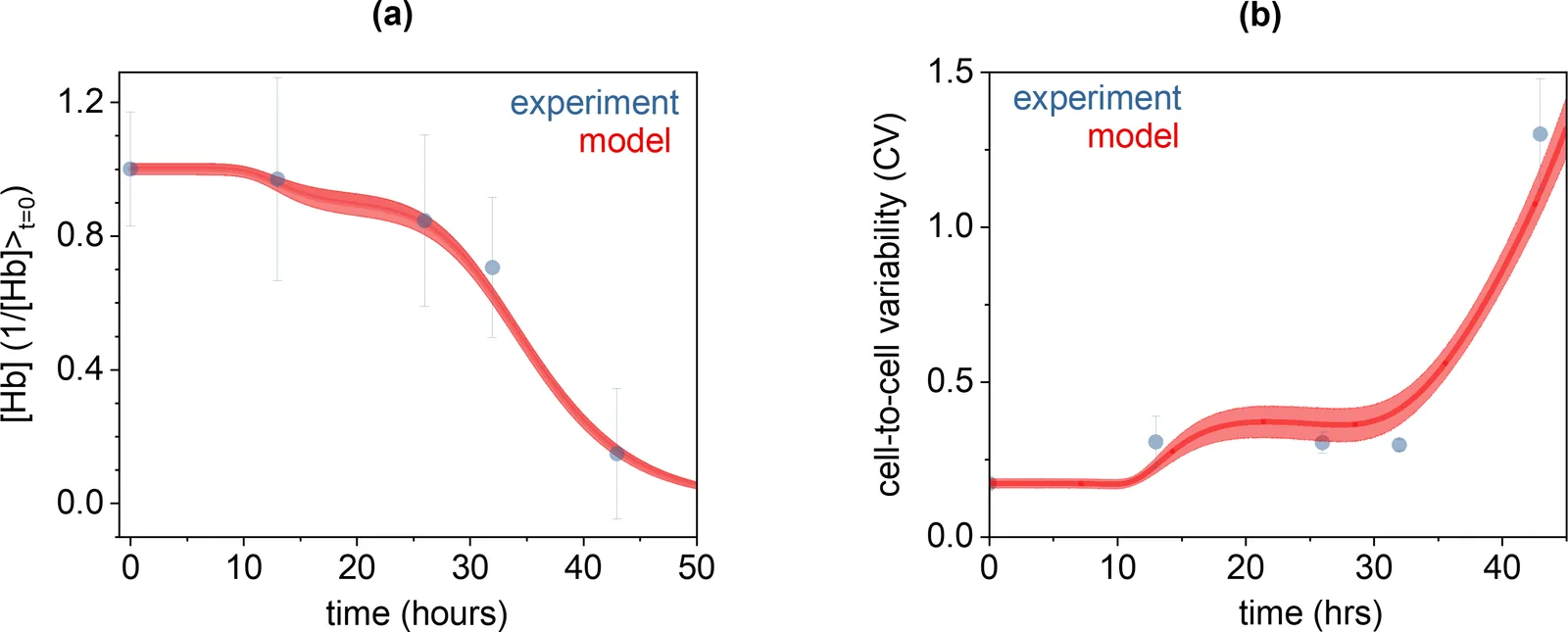
Progressive Hb depletion and increasing cell-to-cell variability during the *Plasmodium falciparum* asexual cycle. (**a**) Dependence of Hb content on asexual *P. falciparum* developmental stage, normalized to the Hb concentration of uninfected RBCs. Data points represent mean ± standard deviation. The thick red line shows the fit of the revised Lotka-Volterra-inspired Hb-consumption model, which incorporates a bimodal distribution of the effective consumption parameter, as described in Materials and Methods. The red-shaded area indicates the 95% interval of the model-predicted mean trajectory, estimated by bootstrap resampling of the simulated trajectory ensemble. (**b**) Cell-to-cell differences in Hb concentration,quantified as the coefficient of variation (CV), as a function of *P. falciparum* developmental stage. Points represent experimental CV data, and error bars denote 95% confidence intervals obtained by nonparametric bootstrapping (10,000 resamples per stage). The thick red line shows the corresponding model-predicted CV trajectory from the same revised bimodal model used in panel **a**, and the red-shaded area indicates the 95% interval of the simulation-derived CV trajectory, estimated by bootstrap resampling of the simulated trajectory ensemble.

We next examined how erythrocyte-to-erythrocyte variability in Hb content (RBC_mass_; Fig. 3a) manifests as a function of parasite age. Plotting the Hb concentration of host erythrocytes against parasite age (Fig. 4a) revealed an overall decline, consistent with the digestion of intraerythrocytic Hb. Both the magnitude and temporal trajectory of the observed Hb decrease are consistent with prior single-cell QPI studies that measured reductions in infected RBC dry mass during *P. falciparum* development (36). Because the cultures were asynchronous, parasite age was approximated by mapping the morphological stages R, LR-ET, T, and Sch to 13, 26, 32, and 43 h post-invasion, respectively (47). Thus, Fig. 4a represents a cross-sectional stage-to-age approximation rather than a synchronized longitudinal time course. Notably, the spread around the mean Hb concentration widened with progression through the 48-h cycle (Fig. 4a). Because these measurements were obtained from an asynchronous population, this broadening reflects both developmental progression and within-stage heterogeneity. Specifically, cell-to-cell differences in Hb concentration (CV) rose from ~17% in uninfected RBCs to ~31% in rings, remained at ~30% during the LR-ET and trophozoite stages, and then increased sharply at the schizont stage to ~131% (Fig. 4b). These data suggest that RBCs from opposite ends of the host Hb content distribution diverge further after invasion. While it is well-established that parasites exhibit distinct stage-specific consumption rates, our findings suggest that pre-existing host cell heterogeneity contributes to this diversity. In this framework, the initial host phenotypic variability is reflected and expanded within the infected population, contributing to the dispersion observed in Hb consumption rates.

With these observations in mind, we developed a minimal model adapting Lotka-Volterra predator-prey dynamics (Materials and Methods), treating intracellular Hb as the prey and parasite biomass as the predator (49, 50). We adopted this two-equation framework (Materials and Methods) because it reflected earlier models in host-microbe systems and provided sufficient explanatory power with only two fitted parameters, thereby limiting the risk of overfitting (51). As further detailed in Materials and Methods, we first quantified the mean decline in host cell Hb using the analytical solution for Hb concentration over time, *y*(*t*), shown in the following equation:


(3) $$y\left(t\right)=F\left(y_{0},t\right):=\frac{y_{0}\cdot \left(1+a\cdot y_{0}\right)}{e^{g\cdot t\left(1+a\cdot y_{0}\right)}+a\cdot y_{0}}.$$

In equation 3, the two lumped parameters *a* and *g* are related to the Hb-to-biomass conversion yield and the effective Hb-consumption term, respectively (see equations 7 and 8 in Materials and Methods). Fitting equation 3 to the stage-ordered mean Hb data reproduced the overall trajectory in Fig. 4a. However, because the experimental data showed an early increase in variability that was not fully captured by a single effective consumption rate, we extended the model to allow for a bimodal distribution of the effective consumption parameter. Specifically, we modeled the infected-cell population as comprising a major subpopulation with a lower effective Hb-consumption rate and a minor subpopulation with a higher effective rate.

To capture increasing cell-to-cell differences in Hb concentration across parasite stages (Fig. 4b), we generated 350 *in silico* Hb decay trajectories using the revised model, with initial Hb levels sampled from the distribution measured in non-infected RBCs. We then computed the coefficient of variation in Hb levels across simulated trajectories and compared with the experimental data. This revised model provided an improved fit to the temporal evolution of both mean Hb levels and their coefficient of variation across parasite development, including the early post-invasion stages (Fig. 4a and b). The fit to the mean Hb trajectory was strong (*R*^2^ = 0.987), and simulations based on the revised formulation also captured the modest early increase in heterogeneity together with the pronounced broadening observed at the schizont stage. Although variability at the first post-invasion time point remains especially sensitive to biological heterogeneity, the updated model captures the overall increase in dispersion substantially better than the previous formulation. Together, these results highlight the central role of Hb variability of the uninfected host in shaping parasite development and suggest that a small subpopulation of infected cells may exhibit elevated effective Hb-consumption dynamics.

## DISCUSSION

Infections by intracellular pathogens are shaped not only by pathogen genetics and environment but also by the phenotypic variability, or noise, of the host cells they invade. While pathogen phenotypic noise has been intensively studied, the contribution of natural, non-genetic host cell variation remains less understood. Here, we have shown that differences in RBC Hb concentration, a property of RBCs that precedes infection, can contribute to variation in Hb consumption among genetically identical *P. falciparum* parasites. This systems-level perspective reframes our understanding of intraerythrocytic development: the host’s phenotypic “landscape” is not mere passive background but an active determinant of parasite development. The progressive amplification of this heterogeneity is an emergent outcome of host-parasite interactions: as parasites consume Hb, the pre-existing host distribution is non-linearly amplified. Although more complex models incorporating stage-specific kinetics or variable conversion efficiencies could offer further refinement, our revised model shows that a parsimonious framework based on host cell heterogeneity together with heterogeneity in effective Hb-consumption dynamics is sufficient to capture the primary distributional broadening observed across the cycle. Because uninfected and infected RBCs were measured as distinct cross-sectional populations, the uninfected-RBC CV is interpreted as baseline host cell heterogeneity that is linked to infected-stage variability through the mathematical model, rather than as a direct single-cell prediction.

We quantified Hb content (RBC_mass_) variability in uninfected RBCs and found a fourfold range (Fig. 3a). This variability partly reflects the strong dependence of total Hb mass on RBC size (Fig. 3c), whereas Hb concentration showed little correlation with cell size (Fig. 3b), indicating that cytosolic Hb packing varies largely independently of cell area. Thus, cell size is a major determinant of total Hb mass but not the sole descriptor of host cell Hb variability. While Hb concentration did not correlate with cell size (Fig. 3b), theory suggests that a fourfold range in Hb concentration can alter Hb activity coefficients by two orders of magnitude, substantially changing the digestible pool available to the parasite (52). After invasion, the Hb concentration variability expanded further, with CV rising from ~17% in RBCs to ~31% in rings (95% bootstrap CI 20%–39%), plateauing around ~30% through LR-ET (27%–34%) and trophozoites (28%–32%), and then increasing dramatically to ~131% in schizonts (109%–151%; Fig. 4b). These confidence intervals emphasize that while ring- and schizont-stage measurements carry greater uncertainty due to lower sample size, the overall trajectory of modest increase early, plateau mid-cycle, and sharp amplification at schizogony remains robust. Bootstrap analysis therefore confirms that the rise in variability is not an artifact of sampling imbalance but reflects a genuine biological pattern of progressive amplification of host-derived heterogeneity across the asexual cycle.

The especially large variability observed at the schizont stage is consistent with cumulative amplification of Hb depletion over the intraerythrocytic cycle. Prior ultrastructural work provides mechanistic context for this stage-dependent Hb acquisition: Elliott et al. identified multiple parasite Hb uptake pathways, including cytostome-derived vesicles, cytostomal tubes, and large Hb-containing vacuoles. Although these ultrastructural measurements are not directly comparable to QPI-derived Hb-mass trajectories, they support the interpretation of Hb depletion as an active, developmentally structured process. (53). This increase is unlikely to reflect technical noise alone, as prior fixed-cell measurements on the same SLIM platform indicated an instrument noise floor corresponding to a density uncertainty of ~1% (54), much smaller than the variability observed here. In addition, the observed broadening is unlikely to arise solely from exclusion of parasite-occupied regions, since schizont-stage host cell estimates were still computed from an average of 4,076 pixels per cell. We focused on this host cytosolic Hb pool because these regions could be identified reliably (36), whereas parasite-occupied regions were less suitable for Hb analysis due to their small size. Finally, to account for unequal sample sizes across parasite stages, we quantified uncertainty in stage means and coefficients of variation using nonparametric bootstrapping (10,000 resamples per stage). Thus, although masking, unequal sample sizes, and instrumental noise may contribute to application-specific uncertainty, they are unlikely to explain the full magnitude of the observed broadening (30).

A Lotka-Volterra model reproduced both the mean Hb decay curve and stage-specific noise (Fig. 4) when we included (i) the variability of starting Hb and (ii) the asynchrony within morphological stages. Thus, our results indicate that pre-existing host cell heterogeneity contributes substantially to the observed dispersion in parasite Hb consumption, while the model further suggests that heterogeneity in effective consumption dynamics may also contribute. Because RBC Hb content (RBC_mass_) is central to this interpretation, we also attempted to establish an additional Hb-content control using mRBCs; however, the mRBC and donor-RBC single-cell Hb-content distributions overlapped substantially, indicating that this preparation did not provide a clean experimental control for isolating Hb content as the sole RBC variable (Fig. S2). Parasite-associated factors, including developmental heterogeneity and inter-parasite communication, may also contribute to variability beyond that captured by the current framework (55, 56).

The pronounced variability in host Hb content that we observed before invasion raises the question of its origins. One possibility is variation in RBC quality due to variable cell age or storage-related conditions, which has previously been shown to modulate parasite behavior, consistent with the interpretation of our results (35). It is also possible that variability at this scale originates during erythropoiesis (57): globin-gene expression increases considerably in polychromatic and orthochromatic erythroblasts (58, 59). This increase continues through enucleation, where protein synthesis relies on pre-existing ribosomes (60). Therefore, stochastic fluctuations (1–8) in globin gene expression, or differential loss of Hb, ribosomes, and mRNA during enucleation could generate the observed Hb content (RBC_mass_) variability in uninfected RBCs (61). Identifying the dominant contributor will require single-cell analyses of marrow precursors, an emerging possibility thanks to recent technical advances (62).

By quantifying and modeling the link between host cell variability and parasite nutrient consumption, our work may open new paths for malaria research and treatment. Integrating host phenotypic noise into within-host models could improve predictions of parasite progression and optimize the timing of activation for heme-dependent antimalarials, including artemisinin, its derivatives, and synthetic ozonides, which rely on host Hb digestion for their parasiticidal activity (30–33, 63). This framework could also offer a mechanistic explanation for patient-to-patient variability in drug responses and infection persistence, even when parasite genetics are similar (56). By using a single donor lot, we were able to isolate the impact of cell-to-cell heterogeneity from donor-to-donor variability. Future studies utilizing multiple donors will be important to assess how host-specific factors might further modulate the quantitative relationships observed here. More broadly, our approach illustrates how single-cell measurements and quantitative models can be combined to reveal emergent properties of host-pathogen systems. Future longitudinal single-cell studies will fully exploit host phenotypic variability as a predictive variable in infectious disease biology.

## MATERIALS AND METHODS

### Microscopy

Images were obtained using a quantitative phase imaging system (Phi Optics), which relies on the spatial light interference microscopy (SLIM) principle (43). This technique involves creating phase images by projecting the back focal plane of a phase contrast objective onto a liquid crystal phase modulator. This modulator alters the optical phase of the light wavefront scattered by the sample in relation to the unscattered light (43). This method allowed us to capture images showing the relative phase delay between the cells and their cytosolic components (scattered wavefront) compared with the background (unscattered wavefront).

The QPI/fluorescence system was built around an inverted Leica DMi8 microscope with phase-contrast and epifluorescence capabilities, an automated *XYZ* stage, and a 63× magnification objective (NA 0.7, PH2, Leica). QPI and epifluorescence images were the only imaging modalities acquired for the cells analyzed here; no separate DIC or conventional brightfield images were collected. Images were captured using the Orca Flash 4.0 scientific CMOS camera (Hamamatsu) with a 6.5-µm pixel size. Acquisition settings included a 50-ms exposure time and a 50-ms refresh rate for the spatial light modulator. This setup provided an optimal balance between stability and temporal resolution, consistent with our previous findings (64). For each experimental condition, 2D images were collected by scanning the stage in the *XY* plane until ~1,130 single-cell observations were obtained. For infected RBCs, parasite-occupied regions were identified from paired QPI-fluorescence images acquired from the same cells and conservatively excluded from the Hb-mass analysis. Residual host cell Hb mass was then calculated from the integrated phase signal across all reliably identifiable host-cytosolic pixels, rather than from selected point measurements. This masking strategy was used to minimize parasite-associated phase contributions while providing a robust estimate of the resolved host-cytosolic Hb pool. In our analysis of parasite counts across different life stages, we observed an imbalance in the distribution, with uninfected cells (*n* = 385), rings (*n* = 70), LR-ET (*n* = 275), trophozoites (*n* = 307), and schizonts (*n* = 94). Because of this, we estimated 95% confidence intervals for mean values and coefficients of variation using nonparametric bootstrapping (10,000 resamples per stage). This approach quantified the uncertainty associated with the smaller ring and schizont groups and ensured robust comparisons across stages.

### Propidium iodide fluorescence imaging and stage classification

For infected RBC samples, propidium iodide (PI; 5 μM) epifluorescence imaging was used to visualize parasite nuclei and support life stage classification. Fixed infected RBCs were incubated with PI for approximately 30 min prior to imaging. No blocking step was used because PI is a direct nucleic-acid stain rather than an antibody-based label, and no post-staining wash was performed before imaging because PI fluorescence is strongly enhanced upon nucleic-acid binding, producing high-contrast nuclear signal suitable for stage classification. Paired QPI and PI epifluorescence images were then acquired from the same cells on the Leica DMi8 microscope described above. As an internal negative control acquired contemporaneously with the experimental images, uninfected RBCs in the same fields were identified by QPI morphology and lacked PI-positive parasite nuclear signal. PI-positive nuclear signal, together with the paired QPI-based parasite morphology, was used to classify parasites into ring, late ring/early trophozoite, trophozoite, and schizont stages. Stage assignment was performed independently by two trained annotators. In cases of disagreement, classifications were resolved by joint review and consensus.

### Mathematical modeling of Hb consumption

To investigate the mechanisms driving the observed variations in Hb consumption among infected RBCs, we developed a mathematical model inspired by Lotka-Volterra predator-prey dynamics (49, 50). This model captures the interaction between Hb concentration and parasite biomass over time. The interaction between Hb concentration (*y*) and parasite biomass (*x*) is described by the following nonlinear system of ordinary differential equations:


(4) $$\frac{dy}{dt}=-c\cdot y\left(t\right)\cdot x\left(t\right)$$

(5) $$\frac{dx}{dt}=d\cdot y\left(t\right)\cdot x\left(t\right)$$

where *y*(*t*) and *x*(*t*) are Hb concentration and parasite biomass, at time *t*, respectively. Here, *c* denotes the per capita consumption rate of Hb, and the ratio *d*/*c* can be interpreted as the conversion of Hb into parasite biomass. This system provides the solution for Hb levels over time given by equation 3. In this context, the lumped parameters *a*, *g* are:


(6, 7) $$a=\frac{\;d}{c\cdot x_{0}},\;g=c\cdot x_{0}$$

Here, *y*_0_ and *x*_0_ are values of *y*(*t*) and *x*(*t*), respectively, at time *t* = 0.

Because the experimental data showed an increase in Hb variability at early time points, we extended the original single-rate formulation to allow heterogeneity in the effective consumption parameter *g*. Specifically, we assumed that a fraction *f* of parasites follows a lower effective consumption rate, *g*_low_, whereas the remaining fraction 1 − *f* follows a higher effective consumption rate, *g*_high_. Under this bimodal formulation, the mean Hb level over time is given by:


(8) $$y\left(t\right)=f\cdot \frac{y_{0}\cdot \left(1+\alpha \cdot y_{0}\right)}{e^{g_{low}\cdot t\left(1+\alpha \cdot y_{0}\right)}+\alpha \cdot y_{0}}+\left(1-f\right)\cdot \frac{y_{0}\cdot \left(1+a\cdot y_{0}\right)}{e^{g_{high}\cdot t\left(1+a\cdot y_{0}\right)}+a\cdot y_{0}}$$

Fitting equation 9 to the data on the decay in average Hb levels over time yielded an excellent fit (*R*^2^ = 0.987), with inferred parameters *g*_low_ ≈ 3.5 × 10^−6^ day^−1^, *g*_high_ ≈ 1.1 × 10^−5^ h^−1^, *f* = 0.9, and the dimensionless ratio *a* · *y*_0_ ≈ 90,820. These parameter estimates suggest that approximately 10% of parasites exhibit an effective Hb consumption rate roughly threefold higher than the baseline subpopulation. For this fitting, we assumed parasite stage R, LR-ET, T, and S correspond to 13, 26, 32, and 43 h, respectively (47). Our minimal model represents the Hb decay within a single erythrocyte over its 48-h infection cycle. As the imaging data represents a cross-sectional snapshot of an asynchronous population at a fixed harvest point, the model assumes a constant distribution of stages and does not explicitly account for the increase in total parasitemia resulting from new invasion events over the course of multiple cycles. To capture transient variability in Hb decay, we generated 350 *in silico* Hb decay trajectories using equation 9, with initial Hb levels (*y*_0_) sampled from the distribution measured in non-infected RBCs. The coefficient of variation in Hb levels over time was then computed across simulated trajectories and compared with the experimental data.

### Samples

#### Human blood preparation

Human RBCs were prepared from a single commercially purchased O+ human blood donor lot (Grifols Bio Supplies, Inc., Los Angeles, CA, USA) immediately after receipt or, at the latest, the following day, and stored at 4°C. After receipt, blood was transferred into sterile 50-mL conical tubes and supplemented with RPMI 1640 (without HEPES) to 50% (vol:vol). The contents were mixed by gently inverting the tubes, followed by centrifugation at 4°C for 10 min at 1,000 × *g*. After centrifugation, the supernatant was aspirated using a glass pipette, with these steps repeated three times to remove white blood cells (WBCs) and proteins. Washed RBCs were transferred to a fresh 50-mL conical tube, mixed with an equal volume of regular RPMI 1640, and stored at 4°C for a maximum of 2 weeks. Washed RBCs were sampled, fixed in 2% paraformaldehyde (overnight at 4°C) and washed (3×) in fresh RPMI 1640.

#### *Plasmodium falciparum* culture

To initiate the culture, frozen stock of *P. falciparum* NF54-infected RBCs was warmed to 37°C and washed three times with decreasing concentrations of NaCl (12%, 1.6%, 0.9%) to remove glycerol. Washed RBCs were then transferred to small flasks. Live parasites were grown at 37°C in complete RPMI-1640 medium supplemented with 10% heat-inactivated human serum, HEPES, L-glutamine, hypoxanthine, and DL-lactic acid with 4.0%–6.0% washed type O+ RBCs prepared as described above. Medium was changed each day, followed by flow of mixed gas (5% CO_2_, 5% O_2_, 90% N_2_) into the flask. Every 2 days, the percentage of infected RBCs was determined using microscopic examination of thin blood films stained with Giemsa (65). At 10%–12% infected RBCs, the parasite culture was split into a new flask for maintenance as above. Prior to [Hb] measurements, parasites were maintained in continuous culture for at least three intraerythrocytic cycles. Samples were harvested for imaging when the culture reached approximately 10%–12% parasitemia. For fixation, infected RBCs were sampled from these flasks, fixed in 2% paraformaldehyde (overnight at 4°C), and washed three times in fresh medium

#### *Ex vivo* manufacturing of red blood cells

CD34^+^ hematopoietic stem and progenitor cells (Charles River, cat. no. CB34C) were thawed in a 37°C water bath, immediately resuspended in 9 mL of expansion medium, and centrifuged at 1,000 rpm for 8 min (66). Cells were seeded in expansion medium consisting of Iscove’s modified Dulbecco’s medium (IMDM) with GlutaMAX (Fisher Scientific, cat. no. 31-980-097), 3% (vol/vol) heat-inactivated human AB serum (Sigma, cat. no. H3667), 2 mg/mL human serum albumin (Irvine Scientific, cat. no. 9988), 10 μg/mL insulin (Sigma, cat. no. I9278), 3 U/mL heparin (Sigma, cat. no. H3149), 0.5 mg/mL transferrin (R&D Systems, cat. no. 2914-HT-001G), 3 U/mL erythropoietin (EPO; PeproTech, cat. no. 100-64), 10 ng/mL stem cell factor (SCF; R&D Systems, cat. no. 255-SC-001MG/CF), and 1 ng/mL interleukin-3 (IL-3; R&D Systems, cat. no. 203-IL-010). Cells were cultured for 8 days at 37°C in a humidified incubator with 5% CO_2_ and maintained at a density of 2 × 10⁵ cells/mL throughout the expansion phase. On day 8, cells were collected by centrifugation at 1,200 rpm for 5 min and resuspended at 3 × 10⁵ cells/mL in day 8–9 differentiation medium. This medium consisted of IMDM with GlutaMAX (Fisher Scientific, cat. no. 31-980-097), 2 mM GlutaMAX (Fisher Scientific, cat. no. 35-050-061), 3% (vol/vol) human AB serum, 2 mg/mL human serum albumin, 10 μg/mL insulin, 3 U/mL heparin, 0.75 mg/mL transferrin, 3 U/mL erythropoietin, 10 ng/mL stem cell factor, 0.04 μM sodium selenite (Sigma, cat. no. S5261), 2 mM sodium pyruvate (Fisher Scientific, cat. no. 11-360-070), 2× MEM amino acids (Sigma, cat. no. M5550), 2× MEM vitamins (Sigma, cat. no. M6895), 2× MEM non-essential amino acids (Sigma, cat. no. M7145), 100 U/mL penicillin–streptomycin (Sigma, cat. no. P0781), and 10 μg/mL gentamicin (Sigma, cat. no. G1272). Beginning on day 8, cultures underwent daily 50% medium changes throughout the differentiation period. On day 10, 50% of the culture medium was replaced with fresh day 10 differentiation medium, which was identical to the day 8–9 formulation except that the transferrin concentration was increased from 0.75 to 1 mg/mL. From days 11 to 15, cultures underwent daily 50% medium changes with day 11–15 differentiation medium, which was identical to the day 10 formulation except that heparin, erythropoietin, stem cell factor, and insulin were omitted. From days 16 to 18, cultures underwent daily 50% medium changes with day 16–18 differentiation medium, which was identical to the day 11–15 formulation, except that sodium selenite was also omitted. Cells were harvested on day 19 and processed for downstream analyses.

### Statistics

The coefficient of variation (CV) was computed in Origin Pro. The 95% confidence intervals of all linear regressions were computed in MATLAB by bootstrapping using the bootci function (*n*  =  10,000 samples).

## Supplementary Material

Reviewer comments

## Data Availability

The raw data supporting the conclusions of this article, including single-cell measurements and mathematical modeling outputs, are openly available at https://doi.org/10.5281/zenodo.21910623.

## SUPPLEMENTAL MATERIAL

Supplemental Figures msystems.00554-26-s0001.docx Figures S1 and S2.

OPEN PEER REVIEW reviewer-comments.pdf An accounting of the reviewer comments and feedback.

ASM does not own the copyrights to Supplemental Material that may be linked to, or accessed through, an article. The authors have granted ASM a non-exclusive, world-wide license to publish the Supplemental Material files. Please contact the corresponding author directly for reuse.
