# Supplementary material for: Non-genetic red blood cell variability can modulate hemoglobin consumption by intracellular Plasmodium falciparum
Source: mSystems. 2026 Sep 4;11(10):e00554-26. doi: 10.1128/msystems.00554-26 (PMC13712753; doi:10.1128/msystems.00554-26)
Supplement: Supplementary file 1 [file reviewer-comments.pdf]

ASM | mSystems

Non-Genetic Red Blood Cell Variability Can Modulate Hemoglobin Consumption by Intracellular *Plasmodium falciparum*

Joseph Perko, Abhyudai Singh, Secilia Lopez, Armando Estrada, Shweta Singh, George Klarmann, Sabrina Tuson, Vincent Ho, Shirley Luckhart, and Andreas Vasdekis

Corresponding Author(s): Andreas Vasdekis, University of Idaho

Review Timeline:

Submission Date:	May 5, 2026
Editorial Decision:	May 26, 2026
Revision Received:	July 27, 2026
Accepted:	July 30, 2026

Editor: Jyot Antani

Reviewer(s): Disclosure of reviewer identity is with reference to reviewer comments included in decision letter(s). The following individuals involved in review of your submission have agreed to reveal their identity: Riette Vanbiljon (Reviewer #3)

Transaction Report:

(Note: With the exception of the correction of typographical or spelling errors that could be a source of ambiguity, letters and reports are not edited. The original formatting of letters and referee reports may not be reflected in this compilation.)

DOI: https://doi.org/10.1128/msystems.00554-26

Re: mSystems00554-26 (**Non-Genetic Red Blood Cell Variability Can Modulate Hemoglobin Consumption by Intracellular *Plasmodium falciparum***)

Dear Prof. Andreas E. Vasdekis:

Thank you for the privilege of reviewing your work. Below you will find my comments, instructions from the mSystems editorial office, and the reviewer comments.

Please address the suggestions from the reviewers to add minor details, either in the main text or in the SI as you see fit.

Please return the manuscript within 60 days; if you cannot complete the modification within this time period, please contact me. If you do not wish to modify the manuscript and prefer to submit it to another journal, notify me immediately so that the manuscript may be formally withdrawn from consideration by mSystems.

Revision Guidelines
To submit your modified manuscript, log into the submission site at https://msystems.msubmit.net/cgi-bin/main.plex. Go to Author Tasks and click the appropriate manuscript title to begin. The information you entered when you first submitted the paper will be displayed; update this as necessary. Note the following requirements:

• Upload point-by-point responses to the issues raised by the reviewers in a file named "Response to Reviewers," NOT in your cover letter.
• Upload a compare copy of the manuscript (without figures) as a "Marked-Up Manuscript" file.
• Upload a clean .DOC/.DOCX version of the revised manuscript and remove the previous version.
• Each figure must be uploaded as a separate, editable, high-resolution file (TIFF or EPS preferred), and any multipanel figures must be assembled into one file.
• Any supplemental material intended for posting by ASM should be uploaded with their legends separate from the main manuscript. You can combine all supplemental material into one file (preferred) or split it into a maximum of 10 files with all associated legends included.

For complete guidelines on revision requirements, see our Submission and Review Process webpage. Submission of a paper that does not conform to guidelines may delay acceptance of your manuscript.

Data availability: ASM policy requires that data be available to the public upon online posting of the article, so please verify all links to sequence records, if present, and make sure that each number retrieves the full record of the data. If a new accession number is not linked or a link is broken, provide mSystems production staff with the correct URL for the record. If the accession numbers for new data are not publicly accessible before the expected online posting of the article, publication may be delayed; please contact production staff (mSystems@asmusa.org) immediately with the expected release date.

Publication Fees: For information on publication fees and which article types are subject to charges, visit our website. If your manuscript is accepted for publication and any fees apply, you will be contacted separately about payment during the production process; please follow the instructions in that e-mail. Arrangements for payment must be made before your article is published.

ASM Membership: Corresponding authors may join or renew ASM membership to obtain discounts on publication fees. Need to upgrade your membership level? Please contact Customer Service at Service@asmusa.org.

The ASM Journals program strives for constant improvement in our submission and publication process. Please tell us how we can improve your experience by taking this quick Author Survey.

Thank you for submitting your paper to mSystems.

Sincerely,
Jyot Antani, PhD

Reviewer #2 (Comments for the Author):

The author's addressed my major concerns with additions to the results and methods clarifying the relative magnitudes of the measurement noise and the cell-to-cell variability. The additions to the figures also improve overall interpretability of the data.

The text additions to the discussion greatly improve the overall clarity of the paper.

Reviewer #3 (Comments for the Author):

In the Methods/Results section: Statistical reporting for r2 values following Pearson's correlation should be Pearson r=, instead of rPearson as the authors report and p-values should be italicized.
The PI staining and microscopy method section was not really written with sufficient detail. Please include details ie. the microscope model used for imaging, staining parameters (how long were the samples stained, were they blocked? Were there any washes? Was there corresponding DIC/brightfield images or negative control for staining?) In addition, since the authors imaged the same cells with QPI imaging, it would be appropriate to include representative images of the PI staining as part of that figure or as supporting information.
Line 180: "host-side" is a very strange term to use in this context, perhaps host cell-related would better convey their point.
Line 198-199: These data suggest that RBCs from opposite ends of the host distribution diverge further after invasion. I believe the authors mean "host hemoglobin content distribution".

Reviewer #4 (Comments for the Author):

The authors have adequately addressed the reviewers' concerns, and their efforts are appreciated. The clarification of language, more detailed methodology, and stronger model have improved the manuscript and produced a clearer study. A minor suggestion: the data in Figure r1 (in the response to reviewers) comparing dRBCs versus mRBCs may be of interest to the broader scientific community. Would it be possible to briefly mention this data and include it in the SI?

The author’s addressed my major concerns with additions to the results and methods clarifying the relative magnitudes of the measurement noise and the cell-to-cell variability. The new additions to the text greatly improve the overall quality of the manuscript.

Reviewer #2

Comments for the Author:

The author's addressed my major concerns with additions to the results and methods clarifying the relative magnitudes of the measurement noise and the cell-to-cell variability. The additions to the figures also improve overall interpretability of the data. The text additions to the discussion greatly improve the overall clarity of the paper.

<u>Response</u>: We thank the Reviewer for their positive assessment of the revised manuscript. We are pleased that previous revisions regarding measurement noise, cell-to-cell variability, figure interpretation, and the Discussion improved the clarity of the manuscript.

Reviewer #3

Comments for the Author:

[R3.1] In the Methods/Results section: Statistical reporting for r^2 values following Pearson's correlation should be Pearson r=, instead of rPearson as the authors report and p-values should be italicized.

Response: We agree and have now corrected the statistical notation throughout the manuscript (lines 172 and 176) so that Pearson correlations are now reported as "Pearson r = …" rather than "$r_{Pearson}$ = …". We have also italicized all reported p-values, including those in the **Results** section and any relevant figure captions.

[R3.2] The PI staining and microscopy method section was not really written with sufficient detail. Please include details ie. the microscope model used for imaging, staining parameters (how long were the samples stained, were they blocked? Were there any washes? Was there corresponding DIC/brightfield images or negative control for staining?) In addition, since the authors imaged the same cells with QPI imaging, it would be appropriate to include representative images of the PI staining as part of that figure or as supporting information.

Response: We agree and have revised the **Results** and **Methods** to provide additional detail on the PI staining and microscopy protocols. We also clarify that imaging was performed on an inverted Leica DMi8 microscope, and that the imaging modalities acquired for the analyzed cells were QPI and PI epifluorescence. In this context, the QPI images provided label-free Hb information for the same cells. We have also added the PI staining parameters, including the 5 µM PI concentration, approximately 30-min staining time, absence of blocking, and absence of post-staining washes prior to imaging. Because PI is a direct nucleic-acid stain and its fluorescence is strongly enhanced upon DNA binding, the nuclear parasite signal was readily distinguished from background. We further clarify that uninfected RBCs present in the same imaging fields, identified by QPI morphology, lacked PI-positive parasite nuclear signal and therefore served as internal same-field negative controls acquired contemporaneously with the experimental images. Finally, we revised the main text and the **Fig. 2** legend to make it explicit that these are paired QPI and PI fluorescence images acquired from make same infected RBCs.

From (line 127): "*Stage classification: To classify the life-cycle stage of each parasite, we complemented the SLIM-QPI quantification of Hb with propidium-iodide fluorescence (FL) of the same infected RBCs. In this way, FL imaging enabled visualization of the parasite nucleus and the determination of parasite's life-cycle stage. Unlike recent QPI developments,[45] the SLIM module incurs polarization-dependent optical losses.[43] To minimize these losses, we collected FL images on an independent optical path equipped with a sensitive sCMOS sensor (**Methods**).*"
To (line 133): "*Stage classification: To classify the life-cycle stage of each parasite, we complemented the SLIM-QPI quantification of Hb with propidium-iodide fluorescence (FL) of the same*

*infected RBCs. In this way, each cell used for stage classification had a corresponding paired QPI image and PI epifluorescence image. The QPI images provided the label-free morphological/transmitted-phase information used for staging; no separate DIC or conventional brightfield images were acquired. FL imaging enabled visualization of the parasite nucleus and determination of the parasite life-cycle stage. Unlike recent QPI developments,[45] the SLIM module incurs polarization-dependent optical losses.[43] To minimize these losses, we collected PI epifluorescence images on an independent optical path equipped with a sensitive sCMOS sensor (**Methods**).*"

From (lines 149): "***Fig. 2*** *displays representative images of P. falciparum parasites at different life-cycle stages from our dataset, illustrating the progressive increase in parasite biomass.*"
To (lines 155): "***Fig. 2*** *displays representative paired QPI and PI fluorescence images of the same infected RBCs at different life-cycle stages from our dataset, illustrating the progressive increase in parasite biomass and the PI-positive nuclear signal used for stage classification.* "

From (lines 361): "*The QPI system was integrated with an inverted microscope (DMi8, Leica) featuring phase contrast and fluorescent imaging capabilities, an automated XYZ stage, and a 63× magnification objective (NA 0.7, PH2, Leica). Images were captured using the Orca Flash 4.0 scientific CMOS camera (Hamamatsu) with a 6.5 µm pixel size. Acquisition settings included a 50 ms exposure time and a 50 ms refresh rate for the spatial light modulator. This setup provided an optimal balance between stability and temporal resolution, consistent with our previous findings. For each experimental condition, 2D images were collected by scanning the stage in the XY plane until ~1130 single-cell observations were obtained. For infected RBCs, parasite-occupied regions were identified from paired QPI-fluorescence images and conservatively excluded from the Hb-mass analysis.*"
To (lines 375): "*The QPI/fluorescence system was built around an inverted Leica DMi8 microscope with phase-contrast and epifluorescence capabilities, an automated XYZ stage, and a 63× magnification objective (NA 0.7, PH2, Leica). QPI and epifluorescence images were the only imaging modalities acquired for the cells analyzed here; no separate DIC or conventional brightfield images were collected. Images were captured using the Orca Flash 4.0 scientific CMOS camera (Hamamatsu) with a 6.5 µm pixel size. Acquisition settings included a 50 ms exposure time and a 50 ms refresh rate for the spatial light modulator. This setup provided an optimal balance between stability and temporal resolution, consistent with our previous findings.[64] For each experimental condition, 2D images were collected by scanning the stage in the XY plane until ~1130 single-cell observations were obtained. For infected RBCs, parasite-occupied regions were identified from paired QPI-fluorescence images acquired from the same cells and conservatively excluded from the Hb-mass analysis.*"

From (lines 382): "*Propidium iodide fluorescence imaging and stage classification*
For infected RBC samples, propidium iodide (PI, 5 µM) fluorescence imaging was used to visualize parasite nuclei and support life-stage classification. Fixed infected RBCs were stained with PI prior to imaging, and paired QPI-fluorescence images were acquired for the same cells. PI-positive nuclear signal, together with QPI-based parasite morphology, was used to classify parasites into ring, late ring/early trophozoite, trophozoite, and schizont stages. Stage assignment was per-

formed independently by two trained annotators. In cases of disagreement, classifications were resolved by joint review and consensus."

<u>To (lines 399)</u>*:* "*Propidium iodide fluorescence imaging and stage classification*
For infected RBC samples, propidium iodide (PI, 5 μM) epifluorescence imaging was used to visualize parasite nuclei and support life-stage classification. Fixed infected RBCs were incubated with PI for approximately 30 min prior to imaging. No blocking step was used because PI is a direct nucleic-acid stain rather than an antibody-based label, and no post-staining wash was performed before imaging because PI fluorescence is strongly enhanced upon nucleic-acid binding, producing high-contrast nuclear signal suitable for stage classification. Paired QPI and PI epifluorescence images were then acquired from the same cells on the Leica DMi8 microscope described above. As an internal negative control acquired contemporaneously with the experimental images, uninfected RBCs in the same fields were identified by QPI morphology and lacked PI-positive parasite nuclear signal. PI-positive nuclear signal, together with the paired QPI-based parasite morphology, was used to classify parasites into ring, late ring/early trophozoite, trophozoite, and schizont stages. Stage assignment was performed independently by two trained annotators. In cases of disagreement, classifications were resolved by joint review and consensus."

<u>From (lines 483)</u>*:* "***Figure 2*** | *Quantitative-phase and fluorescence views of Plasmodium falciparum-infected RBCs over the intra-erythrocytic cycle:* ***(a)*** *QPI and* ***(b)*** *fluorescence maps of infected RBCs at different life cycle stages (R: rings; LR-ET: late rings-early trophozoites; T: trophozoites; Sch: schizonts); scale bars in* ***(a)*** *denote the wavefront distortion (ΔΦ) in radians.* ***(c)*** *Schematic illustrations of idealized morphological references for each stage.*"

<u>To (lines 540)</u>*:* "***Figure 2*** | *Paired quantitative-phase and PI fluorescence views of Plasmodium falciparum-infected RBCs over the intra-erythrocytic cycle:* ***(a)*** *QPI and* ***(b)*** *PI fluorescence maps acquired from the same infected RBCs at different life cycle stages (R: rings; LR-ET: late rings-early trophozoites; T: trophozoites; Sch: schizonts); scale bars in* ***(a)*** *denote the wavefront distortion (ΔΦ) in radians.* ***(c)*** *Schematic illustrations of idealized morphological references for each stage.*"

[R3.3] Line 180: "host-side" is a very strange term to use in this context, perhaps host cell-related would better convey their point.

<u>Response</u>*:* We agree with the Reviewer and have replaced "*host-side variation*" with "*host cell-related variation*" to improve clarity.

[R3.4] Line 198-199: These data suggest that RBCs from opposite ends of the host distribution diverge further after invasion. I believe the authors mean "host hemoglobin content distribution".

<u>Response</u>*:* We agree and have revised the sentence to improve clarity:

<u>From (line 200)</u>*:* "*These data suggest that RBCs from opposite ends of the host distribution diverge further after invasion*".

To (line 210): "*These data suggest that RBCs from opposite ends of the host Hb content distribution diverge further after invasion*".

Reviewer #4

Comments for the Author:

The authors have adequately addressed the reviewers' concerns, and their efforts are appreciated. The clarification of language, more detailed methodology, and stronger model have improved the manuscript and produced a clearer study. A minor suggestion: the data in Figure r1 (in the response to reviewers) comparing dRBCs versus mRBCs may be of interest to the broader scientific community. Would it be possible to briefly mention this data and include it in the SI?

Response: We thank the Reviewer for their assessment and have added this dataset as **Fig. S2** and briefly mention it in the manuscript. As a consequence, we have accordingly revised the author list. All revisions are listed below:

Revised author list: Joseph R. Perko[1], Abhyudai Singh[2], Secilia I. Lopez[3], Armando Estrada[4,5], Shweta Singh[4,5], George J Klarmann[4,5], Sabrina J. Tuson[1], Vincent B. Ho[4,5], Shirley Luckhart[3,7], Andreas E. Vasdekis[1,*]

New (line 313): "*Because RBC Hb content (RBC_{mass}) is central to this interpretation, we also attempted to establish an additional Hb-content control using mRBCs; however, the mRBC and donor-RBC single-cell Hb-content distributions overlapped substantially, indicating that this preparation did not provide a clean experimental control for isolating Hb content as the sole RBC variable (Fig. S2).*"

New (line 487): "*Ex vivo Manufacturing of Red Blood Cells: CD34⁺ hematopoietic stem and progenitor cells (Charles River, Cat. No. CB34C) were thawed in a 37°C water bath, immediately resuspended in 9 mL of expansion medium, and centrifuged at 1,000 rpm for 8 min.[66] Cells were seeded in expansion medium consisting of Iscove's Modified Dulbecco's Medium (IMDM) with GlutaMAX (Fisher Scientific, Cat. No. 31-980-097), 3% (v/v) heat-inactivated human AB serum (Sigma, Cat. No. H3667), 2 mg/mL human serum albumin (Irvine Scientific, Cat. No. 9988), 10 µg/mL insulin (Sigma, Cat. No. I9278), 3 U/mL heparin (Sigma, Cat. No. H3149), 0.5 mg/mL transferrin (R&D Systems, Cat. No. 2914-HT-001G), 3 U/mL erythropoietin (EPO; PeproTech, Cat. No. 100-64), 10 ng/mL stem cell factor (SCF; R&D Systems, Cat. No. 255-SC-001MG/CF), and 1 ng/mL interleukin-3 (IL-3; R&D Systems, Cat. No. 203-IL-010). Cells were cultured for 8 days at 37°C in a humidified incubator with 5% CO_2 and maintained at a density of 2×10^5 cells/mL throughout the expansion phase. On day 8, cells were collected by centrifugation at 1,200 rpm for 5 min and resuspended at 3×10^5 cells/mL in day 8–9 differentiation medium. This medium consisted of IMDM with GlutaMAX (Fisher Scientific, Cat. No. 31-980-097), 2 mM GlutaMAX (Fisher Scientific, Cat. No. 35-050-061), 3% (v/v) human AB serum, 2 mg/mL human serum albumin, 10 µg/mL insulin, 3 U/mL heparin, 0.75 mg/mL transferrin, 3 U/mL erythropoietin, 10 ng/mL stem cell factor, 0.04 µM sodium selenite (Sigma, Cat. No. S5261), 2 mM sodium pyruvate (Fisher Scientific, Cat. No. 11-360-070), 2× MEM amino acids (Sigma, Cat. No. M5550), 2× MEM vitamins*

(Sigma, Cat. No. M6895), 2× MEM non-essential amino acids (Sigma, Cat. No. M7145), 100 U/mL penicillin–streptomycin (Sigma, Cat. No. P0781), and 10 µg/mL gentamicin (Sigma, Cat. No. G1272). Beginning on day 8, cultures underwent daily 50% medium changes throughout the differentiation period. On day 10, 50% of the culture medium was replaced with fresh day 10 differentiation medium, which was identical to the day 8–9 formulation except that the transferrin concentration was increased from 0.75 to 1 mg/mL. From days 11 to 15, cultures underwent daily 50% medium changes with day 11-15 differentiation medium, which was identical to the day 10 formulation except that heparin, erythropoietin, stem cell factor, and insulin were omitted. From days 16 to 18, cultures underwent daily 50% medium changes with day 16–18 differentiation medium, which was identical to the day 11-15 formulation except that sodium selenite was also omitted. Cells were harvested on day 19 and processed for downstream analyses."

New Fig. S2:

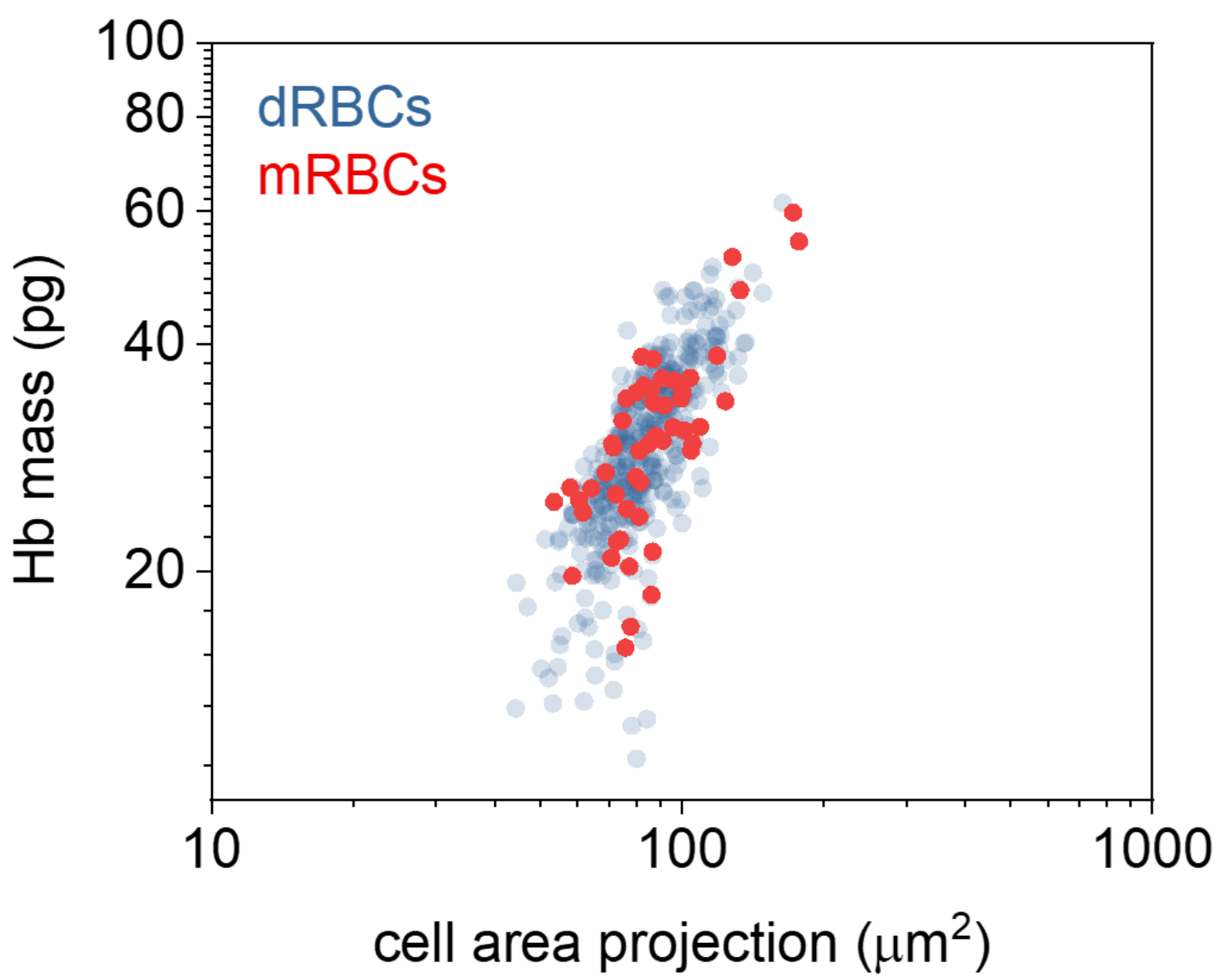

Figure S2 | Single-cell measurements of Hb content in donated red blood cells (dRBCs; *blue*) and manufactured red blood cells (mRBCs; *red*). Each point represents an individual cell. The mRBC population showed Hb-content values that overlapped with, and fell within the range observed for, dRBCs.

Re: mSystems00554-26R1 (**Non-Genetic Red Blood Cell Variability Can Modulate Hemoglobin Consumption by Intracellular *Plasmodium falciparum***)

Dear Prof. Andreas E. Vasdekis:

Your manuscript has been accepted, and I am forwarding it to the ASM production staff for publication. Your paper will first be checked to make sure all elements meet the technical requirements. ASM staff will contact you if anything needs to be revised before copyediting and production can begin. Otherwise, you will be notified when your proofs are ready to be viewed.

Data Availability: ASM policy requires that data be available to the public upon online posting of the article, so please verify all links to sequence records, if present, and make sure that each number retrieves the full record of the data. If a new accession number is not linked or a link is broken, provide production staff with the correct URL for the record. If the accession numbers for new data are not publicly accessible before the expected online posting of the article, publication may be delayed; please contact ASM production staff immediately with the expected release date.

Publication Fees: For information on publication fees and which article types have charges, please visit our website. We have partnered with Copyright Clearance Center (CCC) to collect author charges. If fees apply to your paper, you will receive a message from no-reply@copyright.com with further instructions. For questions related to paying charges through RightsLink, please contact CCC at ASM_Support@copyright.com or toll free at +1-877-622-5543. CCC makes every attempt to respond to all emails within 24 hours.

ASM Membership: Corresponding authors may join or renew ASM membership to obtain discounts on publication fees. Need to upgrade your membership level? Please contact Customer Service at Service@asmusa.org.

Embargo Policy: A press release may be issued as soon as the manuscript is posted on the mSystems Latest Articles webpage. The corresponding author will receive an email with the subject line "ASM Journals Author Services Notification" when the article is available online.

Cover Image Submissions: If you would like to submit a potential Cover Image, please email a file and a short legend to msystems@asmusa.org. Please note that we can only consider images that (i) the authors created or own and (ii) have not been previously published. By submitting, you agree that the image can be used under the same terms as the published article. Image File requirements: TIF/EPS, 7.5 inches wide by 8.25 inches tall (at least 2,250 pixels wide by 2,475 pixels tall), minimum 300 dpi resolution (600 dpi preferred), RGB, and no figure elements, e.g., arrows or panel labels. The legend should be a short description of the image, 1-2 sentences recommended. Please use the ASM Journals Cover Image Sizing Tool (https://journals.asm.org/pb-assets/html/asm-cover-sizing-tool.html) to ensure that your proposed cover image meets our size requirements.

Thank you for submitting your paper to mSystems.

Sincerely,
Jyot Antani, PhD
