# Supplementary material for: Non-genetic red blood cell variability can modulate hemoglobin consumption by intracellular Plasmodium falciparum
Source: mSystems. 2026 Sep 4;11(10):e00554-26. doi: 10.1128/msystems.00554-26 (PMC13712753; doi:10.1128/msystems.00554-26)
Supplement: Supplementary file 2 [file msystems.00554-26-s0001.docx]

**Supplementary Information for**

**Non-Genetic Red Blood Cell Variability Can Modulate Hemoglobin Consumption by Intracellular *Plasmodium falciparum***

Joseph R. Perko^1^, Abhyudai Singh^2^, Secilia I. Lopez^3^, Armando Estrada^4,5^, Shweta Singh^4,5^, Janice M. Moser^6^, Kelly Haupfear^6^, George J. Klarmann^4,5^, Sabrina J. Tuson^1^, Vincent B. Ho^4^, Shirley Luckhart^3,7^, Andreas E. Vasdekis^1, *^

^1^ Department of Physics, University of Idaho, Moscow, ID, 83844, USA.

^2^ Electrical and Computer Engineering, University of Delaware, Newark, DE, USA.

^3^ Department of Biological Sciences, University of Idaho, Moscow, ID, 83844, USA.

^4^ 4D Bio³ Center for Biotechnology, Department of Radiology and Bioengineering, Uniformed Services University of the Health Sciences, Bethesda, MD.

^5^ The Geneva Foundation, Tacoma, WA.

^6^ Sciperio, Inc., Orlando, FL, 32826, USA.

^7^ Department of Entomology, Plant Pathology and Nematology, University of Idaho, Moscow, ID, 83844, USA.

**Supplementary Figure 1**

**Figure S1 |** Representative reconstructed cross-sectional images of RBCs exhibiting **high** [Hb] (top) and **low** [Hb] (bottom). Two representative cells are shown in each category. Pixel intensities are displayed on a normalized scale from 0 to 1.

**Supplementary Figure 2**

**Figure S2 |** Single-cell measurements of Hb content in donated red blood cells (dRBCs; *blue*) and manufactured red blood cells (mRBCs; *red*). Each point represents an individual cell. The mRBC population showed Hb-content values that overlapped with, and fell within the range observed for, dRBCs.
